# Japanese founder duplications/triplications involving *BHLHA9* are associated with split-hand/foot malformation with or without long bone deficiency and Gollop-Wolfgang complex

**DOI:** 10.1186/s13023-014-0125-5

**Published:** 2014-10-21

**Authors:** Eiko Nagata, Hiroki Kano, Fumiko Kato, Rie Yamaguchi, Shinichi Nakashima, Shinichiro Takayama, Rika Kosaki, Hidefumi Tonoki, Seiji Mizuno, Satoshi Watanabe, Koh-ichiro Yoshiura, Tomoki Kosho, Tomonobu Hasegawa, Mamori Kimizuka, Atsushi Suzuki, Kenji Shimizu, Hirofumi Ohashi, Nobuhiko Haga, Hironao Numabe, Emiko Horii, Toshiro Nagai, Hiroshi Yoshihashi, Gen Nishimura, Tatsushi Toda, Shuji Takada, Shigetoshi Yokoyama, Hiroshi Asahara, Shinichiro Sano, Maki Fukami, Shiro Ikegawa, Tsutomu Ogata

**Affiliations:** Department of Pediatrics, Hamamatsu University School of Medicine, Hamamatsu, 431-3192 Japan; Laboratory of Bone and Joint Diseases, Center for Integrative Medical Sciences, RIKEN, Tokyo, Japan; Department of Orthopedic Surgery, Tokyo, Japan; Division of Medical Genetics, National Center for Child Health and Development, Tokyo, Japan; Section of Clinical Genetics, Department of Pediatrics, Tenshi Hospital, Sapporo, Japan; Department of Pediatrics, Central Hospital, Aichi Human Service Center, Kasugai, Japan; Department of Human Genetics, Nagasaki University Graduate School of Biomedical Sciences, Nagasaki, Japan; Department of Medical Genetics, Shinshu University School of Medicine, Matsumoto, Japan; Department of Pediatrics, Keio University School of Medicine, Tokyo, Japan; Department of Orthopedics, National Rehabilitation Center for Disabled Children, Tokyo, Japan; Division of Medical Genetics, Saitama Children’s Medical Center, Saitama, Japan; Department of Rehabilitation Medicine, University of Tokyo, Tokyo, Japan; Department of Genetic Counseling, Graduate School of Humanities and Sciences, Ochanomizu University, Tokyo, Japan; Department of Orthopedic Surgery, Japanese Red Cross Nagoya Daiichi Hospital, Nagoya, Japan; Department of Pediatrics, Dokkyo Medical University Koshigaya Hospital, Koshigaya, Japan; Division of Medical Genetics, Tokyo, Japan; Department of Pediatric Imaging, Tokyo Metropolitan Children’s Medical Center, Tokyo, Japan; Division of Neurology/Molecular Brain Science, Kobe University Graduate School of Medicine, Kobe, Japan; Department of Systems Biomedicine, National Research Institute for Child Health and Development, Tokyo, Japan; Department of Systems Biomedicine, Graduate School of Medical and Dental Sciences, Tokyo Medical and Dental University, Tokyo, Japan; Department of Molecular Endocrinology, National Research Institute for Child Health and Development, Tokyo, Japan; Present address: Laboratory of Metabolism Center for Cancer Research, National Cancer Institute, Bethesda, MD USA

**Keywords:** *BHLHA9*, Split-hand/foot malformation, Long bone deficiency, Gollop-Wolfgang complex, Expressivity, Penetrance, Susceptibility, Japanese founder copy number gain

## Abstract

**Background:**

Limb malformations are rare disorders with high genetic heterogeneity. Although multiple genes/loci have been identified in limb malformations, underlying genetic factors still remain to be determined in most patients.

**Methods:**

This study consisted of 51 Japanese families with split-hand/foot malformation (SHFM), SHFM with long bone deficiency (SHFLD) usually affecting the tibia, or Gollop-Wolfgang complex (GWC) characterized by SHFM and femoral bifurcation. Genetic studies included genomewide array comparative genomic hybridization and exome sequencing, together with standard molecular analyses.

**Results:**

We identified duplications/triplications of a 210,050 bp segment containing *BHLHA9* in 29 SHFM patients, 11 SHFLD patients, two GWC patients, and 22 clinically normal relatives from 27 of the 51 families examined, as well as in 2 of 1,000 Japanese controls. Families with SHFLD- and/or GWC-positive patients were more frequent in triplications than in duplications. The fusion point was identical in all the duplications/triplications and was associated with a 4 bp microhomology. There was no sequence homology around the two breakpoints, whereas rearrangement-associated motifs were abundant around one breakpoint. The rs3951819-*D17S1174* haplotype patterns were variable on the duplicated/triplicated segments. No discernible genetic alteration specific to patients was detected within or around *BHLHA9*, in the known causative SHFM genes, or in the exome.

**Conclusions:**

These results indicate that *BHLHA9* overdosage constitutes the most frequent susceptibility factor, with a dosage effect, for a range of limb malformations at least in Japan. Notably, this is the first study revealing the underlying genetic factor for the development of GWC, and demonstrating the presence of triplications involving *BHLHA9*. It is inferred that a Japanese founder duplication was generated through a replication-based mechanism and underwent subsequent triplication and haplotype modification through recombination-based mechanisms, and that the duplications/triplications with various haplotypes were widely spread in Japan primarily via clinically normal carriers and identified via manifesting patients. Furthermore, genotype-phenotype analyses of patients reported in this study and the previous studies imply that clinical variability is ascribed to multiple factors including the size of duplications/triplications as a critical factor.

**Electronic supplementary material:**

The online version of this article (doi:10.1186/s13023-014-0125-5) contains supplementary material, which is available to authorized users.

## Introduction

Split-hand/foot malformation (SHFM), also known as ectrodactyly, is a rare limb malformation involving the central rays of the autopod [1,2]. It presents with median clefts of the hands and feet, aplasia/hypoplasia of the phalanges, metacarpals, and metatarsals, and syndactyly. SHFM results from failure to maintain the central portion of the apical ectodermal ridge (AER) in the developing autopod [1,2]. SHFM is divided into two forms: a non-syndromic form with limb-confined manifestations and a syndromic form with extra-limb manifestations [2]. Furthermore, non-syndromic SHFM can occur as an isolated abnormality confined to digits (hereafter, SHFM refers to this type) or in association with other limb abnormalities as observed in SHFM with long bone deficiency (SHFLD) usually affecting the tibia and in Gollop-Wolfgang complex (GWC) characterized by femoral bifurcation [1,2]. Both syndromic and non-syndromic forms are associated with wide expressivity and penetrance even among members of a single family and among limbs of a single patient [2].

SHFM and SHFLD are genetically heterogeneous conditions reviewed in ref. [2]. To date, SHFM has been identified in patients with heterozygous deletions or translocations involving the *DLX5*–*DLX6* locus at 7q21.2–21.3 (SHFM1) [3] (*DLX5* mutations have been detected recently), heterozygous duplications at 10q24 (SHFM3), heterozygous mutations of *TP63* at 3q27 (SHFM4), heterozygous deletions affecting *HOXD* cluster at 2q31 (SHFM5), and biallelic mutations of *WNT10B* at 12q31 (SHFM6); in addition, SHFM2 has been assigned to Xq26 by linkage analyses in a large Pakistani kindred [2]. Similarly, a genomewide linkage analysis in a large consanguineous family has identified two SHFLD susceptibility loci, one at 1q42.2–q43 (SHFLD1) and the other at 6q14.1 (SHFLD2); furthermore, after assignment of another SHFLD locus to 17p13.1–13.3 [4], duplications at 17p13.3 (SHFLD3) have been found in patients with SHFLD reviewed in ref. [2]. However, the GWC locus (loci) remains unknown at present.

The duplications at 17p13.3 identified to date are highly variable in size, and harbor *BHLHA9* as the sole gene within the smallest region of overlap [5-9]. *Bhlha9*/*bhlha9* is expressed in the limb bud mesenchyme underlying the AER in mouse and zebrafish embryos, and *bhlha9* knockdown has resulted in shortening of the pectoral fins in zebrafish [6]. Furthermore, *BHLHA9*-containing duplications have been identified not only in patients with SHFLD but also in those with SHFM and clinically normal family members [4-10]. These findings argue for a critical role of *BHLHA9* duplication in the development of SHFM and SHFLD, with variable expressivity and incomplete penetrance.

In this study, we report on *BHLHA9*-containing duplications/triplications with an identical fusion point and various haplotype patterns that were associated with a range of limb malformations including GWC, and discuss on characteristic clinical findings, genomic basis of Japanese founder copy number gains, and underlying factors for phenotypic variability.

## Materials and methods

### Patients/subjects

**Figure 1:**
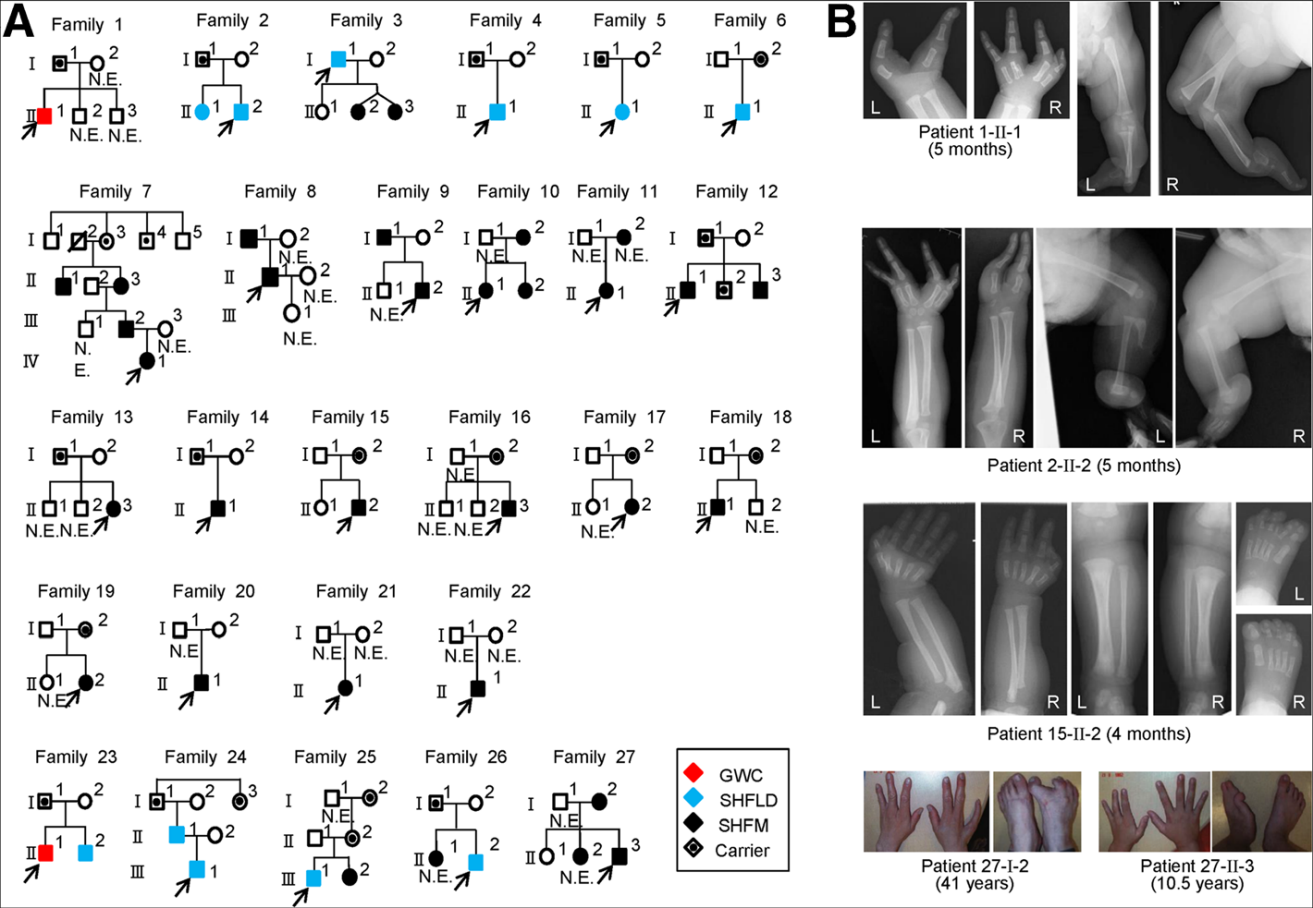
**Clinical summary. A**. Pedigrees of 27 Japanese families with duplications (families 1–22) and triplications (families 23–27) of a ~200 kb region involving *BHLHA9*. The duplications/triplications are associated with GWC, SHFLD, SHFM, or normal phenotype (carriers). N.E.: Not examined molecularly. **B**. Representative clinical findings. Each patient is indicated by a family-generation-individual style and corresponds to the patient/subject shown in Figure 1A and Additional file 5. The top panel: GWC with right bifid femur; the second panel: SHFLD with bilateral tibial deficiencies, the third panel: SHFM with polydactyly; and the bottom panel: SHFM.

We studied 68 patients with SHFM (n = 55), SHFLD (n = 11), or GWC (n = 2), as well as 60 clinically normal relatives, from 51 Japanese families; the pedigrees of 27 of the 51 families and representative clinical findings are shown in Figure 1. All the probands 1–51 had a normal karyotype. Southern blot analysis for SHFM3 locus had been performed in 28 probands with SHFM, indicating 10q24 duplications in two of them [11]. Clinical features including photographs and roentgenograms of a proband with GWC and his brother with SHFLD (family 23 in Figure 1A) were as described previously [12]. The residencies of families 1–51 were widely distributed throughout Japan.

### Ethical approval and samples

This study was approved by the Institutional Review Board Committees of Hamamatsu University School of Medicine, RIKEN, and National Center for Child Health and Development, and was performed using peripheral leukocyte samples after obtaining written informed consent for the molecular analysis and the publication of genetic and clinical data after removing information for personal identification (e.g., name, birthday, and facial photograph) from the adult subjects (³ 20 years) or from the parents of the child subjects (below 20 years). Furthermore, informed assent was also obtained from child subjects between 6–20 years.

### Samples and primers

The primers utilized in this study are summarized in Additional file 1.

### Molecular studies

Sanger sequencing, fluorescence *in situ* hybridization (FISH), microsatellite genotyping, Southern blotting, and bisulfite sequencing-based methylation analysis were performed by the standard methods, as reported previously [13]. Quantitative real-time PCR (qPCR) analysis was carried out by the SYBR Green methods on StepOnePlus system, using *RNaseP* as an internal control (Life Technologies). Genomewide oligonucleotide-based array comparative genomic hybridization (CGH) was performed with a catalog human array (4 × 180 K format, ID G4449A) according to the manufacturer’s instructions (Agilent Technologies), and obtained copy number variants/polymorphisms were screened with Agilent Genomic Workbench software using the Database of Genomic Variants (http://dgv.tcag.ca/dgv/app/home). Sequencing of a long region encompassing *BHLHA9* was performed with the Nextera XT kit on MiSeq (Illumina), using SAMtools v0.1.17 software (http://samtools.sourceforge.net/). Exome sequencing was performed as described previously [14].

### Assessment of genomic environments around the fusion points

Repeat elements around the fusion point were searched for using Repeatmasker (http://www.repeatmasker.org). Rearrangement-inducing DNA features were investigated for 300 bp regions at both the proximal and the distal sides of each breakpoint, using GEECEE (http://emboss.bioinformatics.nl/cgi-bin/emboss/geecee) for calculation of the average GC content, PALINDROME (http://mobyle.pasteur.fr/cgi-bin/portal.py#forms::palindrome) and Non-B DB (http://nonb.abcc.ncifcrf.gov) for the examination of the palindromes and non-B (non-canonical) structures, and Fuzznuc (http://emboss.bioinformatics.nl/cgi-bin/emboss/fuzznuc) for the assessment of rearrangement-associated sequence motifs and tri/tetranucleotides [15-20]. For controls, we examined 48 regions of 600 bp long selected at an interval of 1.5 Mb from the entire chromosome 17.

### Statistical analysis

The statistical significance of the frequency was analyzed by the two-sided Fisher’s exact probability test.

## Results

### Sequence analysis of the known causative/candidate genes

We performed direct sequencing for the previously known causative genes (*DLX5*, *TP63*, and *WNT10B*) reviewed in ref. [2] in the probands 1–51. Although no pathologic mutation was identified in *DLX5* and *TP63*, the previously reported homozygous missense mutation of *WNT10B* (c.944C > T, p.R332W) [21] was detected in the proband 48 with SHFM who was born to healthy consanguineous parents heterozygous for this mutation. In addition, while no variation was detected in *DLX5* and *WNT10B*, rs34201045 (4 bp insertion polymorphism) in *TP63* [21] was detected with an allele frequency of 61%.

We also examined *BHLHA9*, because gain-of-function mutations of *BHLHA9* as well as *BHLHA9*-harboring duplications may lead to limb malformations. No sequence variation was identified in the 51 probands.

### Array CGH analysis

Array CGH analysis was performed for the probands 1–51, showing increased copy numbers at 17p13.3 encompassing *BHLHA9* (SHFLD3) in the probands 1–27 from families 1–27 (Figure 1A). Furthermore, heterozygous duplications at 10q24 (SHFM3) were detected in the probands 49–51, i.e., a hitherto unreported patient with paternally inherited SHFM (his father also had the duplication) and the two patients who had been indicated to have the duplications by Southern blot analysis [11]. No copy number alteration was observed at other SHFM/SHFLD loci in the probands 1–27 and 49–51. In the remaining probands 28–48, there was no copy number variation that was not registered in the Database of Genomic Variants.

### Identical fusion points in *BHLHA9*-containing duplications/triplications

**Figure 2:**
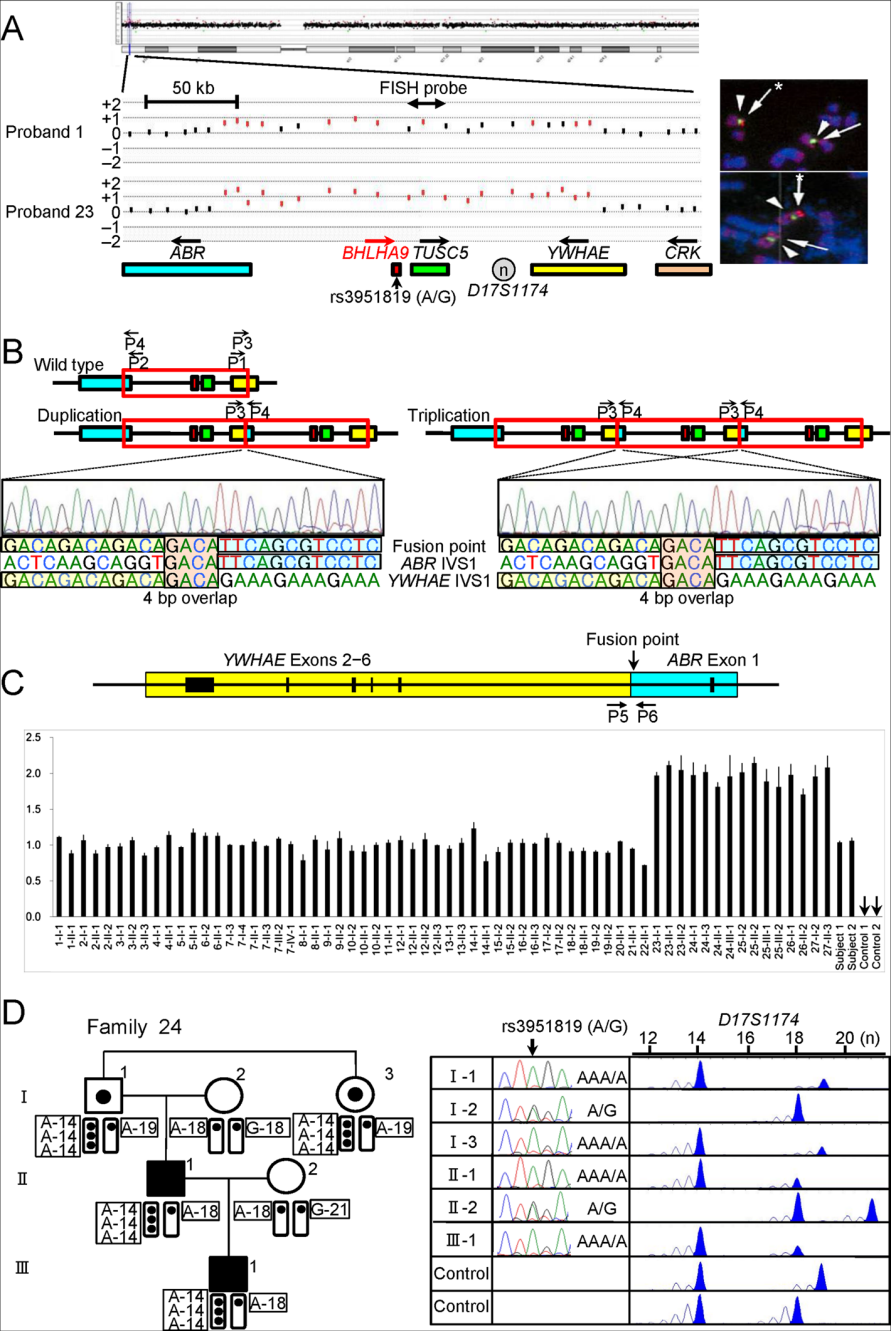
**Identification and characterization of the duplications/triplications involving** ***BHLHA9*** **at chromosome 17p13.3. A**. Array CGH and FISH analyses in proband 1 and proband 23 with GWC. In array CGH analysis, the black and the red dots denote the normal and the increased copy numbers, respectively. Since the log2 signal ratios for a ~200 kb region encompassing *BHLHA9* are around +0.5 in the proband 1 and around +1.0 in the proband 23, this indicates the presence of three and four copies of this region in the two probands, respectively. In FISH analysis, two red signals with an apparently different density are detected by the 8,289 bp PCR probe (the stronger signals are indicated with asterisks). The green signals derive from an internal control probe (CEP17). The arrows on the genes show transcriptional directions. Rs3951819 (A/G) resides within *BHLHA9*. **B**. Determination of the fusion point. The fusion has occurred between intron 1 of *ABR* and intron 1 of *YWHAE*, and is associated with a 4 bp (GACA) microhomology. P1–P4 show the position of primers. **C**. Quantitative real-time PCR analysis. The upper part denotes the fusion point. P5 & P6 show the position of primers. The lower part shows the copy number of the fusion point in patients/subjects with duplications/triplications (indicated by a family-generation-individual style corresponding to that in Figure 1 and Additional file 5). Subject-1 and subject-2 denote the two control subjects with the duplication, and control-1 and control-2 represent normal subjects without the duplication/triplication. **D**. The rs3951819 (A/G SNP)–*D17S1174* (CA repeat number) haplotype patterns in family 24. Assuming no recombination between rs3951819 and *D17S1174*, the haplotype patterns of the family members are determined as shown here. The haplotype patterns of the remaining families have been interpreted similarly.

The array CGH indicated that the increased copy number regions at 17p13.3 were quite similar in the physical size in the probands 1–27 and present in three copies in the probands 1–22 and in four copies in the probands 23–27 (Figure 2A). Thus, FISH analysis was performed using 8,259 bp PCR products amplified from this region, showing two signals with a different intensity that was more obvious in the probands 23–27 (Figure 2A).

We next determined the fusion points of the duplications/triplications (Figure 2B). PCR products of 2,195 bp long were obtained with P1/P2 primers in the probands 1–27, and the fusion point was determined by direct sequencing for 418 bp PCR products obtained with P3/P4 primers. The fusion point was identical in all the probands 1–27; it resided on intron 1 of *ABR* and intron 1 of *YWHAE*, and was associated with a 4 bp microhomology.

Then, we performed qPCR analysis for a 214 bp region harboring the fusion point, using P5/P6 primers (Figure 2C and Additional file 2). The fusion point was present in a single copy in the probands 1–22 and in two copies in the probands 23–27. The results showed that the identical genomic segment harboring *BHLHA9* was tandemly duplicated in the probands 1–22 and triplicated in the probands 23–27. According to GRCh37/hg19 (http://genome.ucsc.edu/), the genomic segment was 210,050 bp long.

We also performed array CGH and qPCR for the fusion point in 15 patients other than the probands and 47 clinically normal relatives from the 27 families (Figures 1 and 2C). The duplications/triplications were identified in all the 15 patients. Thus, in a total of 42 patients, duplications/triplications were found in 29 SHFM patients, 11 SHFLD patients, and two GWC patients. Furthermore, the duplications/triplications were also present in 22 of the 47 clinically normal relatives. In particular, they were invariably identified in either of the clinically normal parents when both of them were examined; they were also present in other clinically normal relatives in families 7, 12, 24, and 25.

Since the above data indicated the presence of duplications/triplications in clinically normal subjects, we performed qPCR for the fusion point in 1,000 Japanese controls. The fusion point was detected in a single copy in two subjects (Subjects 1 and 2 in Figure 2C). We also performed array CGH in 200 of the 1,000 controls including the two subjects, confirming the duplications in the two subjects and lack of other copy number variations, including deletions involving *BHLHA9*, which were not registered in the Database of Genomic Variants in the 200 control subjects. The frequency of duplications/triplications was significantly higher in the probands than in the control subjects (27/51 vs. 2/1,000, *P* = 3.5 × 10^−37^).

### Various haplotype patterns on the duplicated/triplicated segments

We carried out genotyping for rs3951819 (A/G SNP on *BHLHA9*) and *D17S1174* (CA repeat microsatellite locus) on the genomic segment subjected to duplications/triplications (Figure 2A), and determined rs3951819-*D17S1174* haplotype patterns. Representative results are shown in Figure 2D, and all the data are available on request. Various haplotype patterns were identified on the single, the duplicated, and the triplicated segments, and the [A-14] haplotype was most prevalent on the duplicated/triplicated segments (Table 1). While the distribution of CA repeat lengths on the single segments was discontinuous, similar discontinuous distribution was also observed in the Japanese general population (see Additional file 3).

**Table 1:** **The rs3951819 (A/G SNP) –** ***D17S1174*** **(CA repeat number) haplotype**

**Patterns of the 210,050 bp segment subjected to copy number gains**	
**Haplotype pattern**	**Family**
<Single segment>	
[A-14]	1, 5, 9, 15, 17, 19, 23, 26
[A-16]	12
[A-18]	3, 14, 15, 24, 25, 26
[A-19]	2, 6, 13, 19, 20, 24, 25, 27
[A-21]	5, 23
[G-12]	17
[G-14]	2, 3, 6, 12, 13, 19, 26
[G-18]	3, 5, 17, 18, 24, 25
[G-19]	9, 12, 18, 20, 25
[G-21]	1, 9, 19, 24, 27
[A-14] or [G-14]	16
[A-18] or [G-18]	4
[A-19] or [G-19]	4
[A-21] or [G-21]	16
<Duplicated segments>	
[A-14] + [A-14]	5, 12, 13, 14, 15, 20
[A-14] + [A-18]	1
[A-14] + [G-18] or [G-14] + [A-18]	2, 3, 4, 6, 9, 16, 17
[A-14] + [G-18] or [A-14] + [G-19]	18
[A-14] + [G-14] or [G-14] + [G-14]	19
<Triplicated segments>	
[A-14] + [A-14] + [A-14]	23, 24
[A-14] + [A-14] + [G-14]	25
[A-14] + [A-19] + [A-19]	26
[A-14] + [G-18] + [G-18] or [G-14] + [A-18] + [G-18]	27

The haplotype patterns written in the left column have been detected in at least one patient/subject in the families described in the right column.

Genotyping could not be performed in several patients/subjects who had been repeatedly examined previously, because of the extremely small amount of DNA samples that were virtually used up in the sequencing and array CGH analyses.

### Genomic environments around the breakpoints

The breakpoint on *YWHAE* intron 1 resided on a simple *Alu* repeat sequence, and that on *ABR* intron 1 was present on a non-repetitive sequence. There was no low copy repeat around the breakpoints. Comparison of the frequencies of known rearrangement-inducing DNA features between 600 bp sequences around the breakpoints and those of 48 regions selected at an interval of 1.5 Mb from chromosome 17 revealed that palindromes, several types of non-B DNA structures, and a rearrangement-associated sequence motif were abundant around the breakpoint on *YWHAE* intron 1 (see Additional file 4).

### Clinical findings of families 1–27

Clinical assessment revealed several notable findings. First, duplications/triplications were associated with SHFM, SHFLD, GWC, or normal phenotype, with inter- and intra-familial clinical variability (Figure 1A). Second, in the 42 patients, split hand (SH) was more prevalent than split foot (SF) (41/42 vs. 17/42, *P* = 6.2 × 10^−9^), and long bone defect (LBD) was confined to lower extremities (0/42 vs. 13/42, *P* = 4.1 × 10^−5^) (Table 2 and Additional file 5). Third, there was no significant sex difference in the ratio between patients with limb malformations and patients/carriers with duplications/triplications (26/38 in males vs. 16/26 in females, *P* = 0.60) (Table 2 and Additional file 5). Fourth, the ratio of LBD positive families was significantly higher in triplications than in duplications (4/5 vs. 16/22, *P* = 0.047) (Figure 1A and Table 2). Fifth, while the duplications/triplications were transmitted from patients to patients, from carriers to patients, and from a carrier to a carrier (from I-1 to II-2 in family 12), transmission from a patient to a carrier was not identified (Figure 1A); it should be pointed out, however, that molecular analysis in a clinically normal child born to an affected parent was possible only in a single adult subject (II-1 in family 27), and that molecular analysis in clinically normal children <20 years old was possible only in two subjects (II-2 in family 12 and II-1 in family 15). Lastly, limb malformation was inherited in an apparently autosomal dominant manner (from patients to patients), or took place as an apparently *de novo* event or as an apparently autosomal recessive trait (from clinically normal parents to a single or two affected children) (Figure 1A).

**Table 2:** **Summary of clinical findings in patients/carriers with duplications/triplications involving** ***BHLHA9***

	**SHFM (+) patients**			**LBD (+) patients**			Patient ratio*			**LBD (+) families**		
	**SH**	**SF**	***P*** **-value**	**U-LBD**	**L-LBD**	***P*** **-value**	**Male**	**Female**	***P*** **-value**	**Trip**	**Dup**	***P*** **-value**
This study	41/42	17/42	6.2 × 10^−9^	0/42	13/42	4.1 × 10^−5^	26/38	16/26	0.60	4/5	16/22	0.047
Previous studies	63/84	23/84	8.6 × 10^−10^	11/91	42/91	5.7 × 10^−7^	68/114	31/79	5.7 × 10^−3^	…	…	…
Sum	104/126	40/126	1.1 × 10^−16^	11/133	55/133	3.0 × 10^−10^	94/152	47/105	7.6 × 10^−3^	…	…	…

SHFM: split-hand/foot malformation; SH: split hand; SF: split foot; LBD: long bone deficiency; U: upper; L: lower; Trip: triplication; and Dup: duplication.

In the previous studies, patients without detailed phenotypic description and those of unknown sex have been excluded (3–9).

*The ratio between patients with limb malformations and patients/carriers with duplications/triplications, i.e. the number of patients over the number of patients plus carriers.

### Attempts to identify a possible modifier(s)

The variable expressivity and incomplete penetrance in families 1–27 suggest the presence of a possible modifier(s) for the development of limb malformations. Thus, we performed further molecular studies in patients/subjects in whom DNA samples were still available, and compared the molecular data between patients with SHFM and those with SHFLD for the assessment of variable expressivity and between SHFM, SHFLD, or total patients and carriers for the evaluation of incomplete penetrance.

We first examined the possibility that the modifier(s) resides within or around *BHLHA9* (see Additional file 6). There was no *BHLHA9* mutation in all the 21 examined probands with SHFM, SHFLD, or GWC, as described in the section of “Sequence analysis of the known causative/candidate genes”. The rs3951819 A/G SNP pattern on the duplicated/triplicated segments was apparently identical between patients and carriers (e.g. Figure 2D), and the frequency of A/G allele on the normal chromosome 17 was similar between SHFM and SHFLD patients and between SHFM, SHFLD, or total patients and carriers (see Additional file 7). The results of other known SNPs on *BHLHA9* (rs185242872, rs18936498, and rs140504068) were not informative, because of absence or extreme rarity of minor alleles. Furthermore, in SHFM families 7, 12, and 18, sequencing of a 7,406 bp region encompassing *BHLHA9* and Southern blot analysis using five probes and *Mfe*I-, *Ssp*I-, and *Sac*I-digested genomic DNA revealed no variation specific to the patients, and methylation analysis for a CpG rich region at the upstream of *BHLHA9* delineated massive hypomethylation in all the patients/carriers examined.

Next, we examined the possibility that a variant(s) of known causative genes constitutes the modifier(s). Since rs34201045 in *TP63* was identified in the mutation analysis, we compared rs34201045 genotyping data between the 27 probands and the 15 carriers. The allele and genotype frequencies were similar between SHFM and SHFLD patients and between SHFM, SHFLD, or total patients and carriers (see Additional file 8).

We finally performed exome sequencing in SHFM families 13 and 17–19. However, there was no variation specific to the patients. In addition, re-examination of the genomewide array CGH data showed no discernible copy number variation specific to the patients.

## Discussion

### *BHLHA9* overdosage and clinical characteristics

We identified duplications/triplications of a ~ 200 kb genomic segment involving *BHLHA9* at 17p13.3 in 27 of 51 families with SHFM, SHFLD, or GWC. To our knowledge, this is the first study revealing the underlying genetic factor for the development of GWC, and demonstrating the presence of triplications involving *BHLHA9* that were suggested but not confirmed in the previous studies [5,9]. Furthermore, this study indicates that *BHLHA9*-containing duplications/triplications are the most frequent underlying factor for the development of limb malformations at least in Japan. Notably, SHFLD and GWC with LBD were significantly more frequent in patients with triplications than in those with duplications, and the duplications/triplications were identified in clinically normal familial members and in the general population. These findings imply that increased *BHLHA9* copy number constitutes a strong susceptibility, rather than a causative, factor with a dosage effect for the development of a range of limb malformations. Since *Bhlha9* is expressed in the developing ectoderm adjacent to the AER rather than the AER itself in mouse embryos [6], *BHLHA9* appears to play a critical role in the limb development by interacting with the AER. While the duplications/triplications identified in this study included *TUSC5* and generated an *ABR*-*YWHAE* chimeric gene (Figure 2C), *TUSC5* duplication and the chimeric gene formation are not common findings in the previously reported patients with duplications at 17p13.3 and SHFM and/or SHFLD [5-9]. In addition, none of *Tusc5*, *Abr*, and *Ywhae* is specifically expressed in the developing mouse limb buds [22] (A Transcriptome Atlas Database for Mouse Embryo of Eurexpress Project, http://www.eurexpress.org/ee/project/).

Several clinical findings are noteworthy in patients/subjects with duplications/triplications. First, SH was more frequent than SF in this study as well as in the previous studies, and LBD was confined to lower extremities in this study and was more frequent in lower extremities than in upper extremities in the previous studies (Table 2) [4-10]. This implies that *BHLHA9* overdosage exerts differential effects on the different parts of limbs. Second, while limb malformations were similarly identified between males and females in this study, they were more frequently observed in males than in females in the previous studies (Table 2) [4-10]. In this regard, it has been reported that testosterone influences the digital growth pattern as indicated by the lower second to fourth digit length ratio in males than in females [23-25], and that Caucasian males have higher serum testosterone values and lower second to fourth digit length ratios than Oriental males [26,27]. Such testosterone effects on the digital growth pattern with ethnic difference may explain why male dominant manifestation was observed in the previous studies primarily from Caucasian countries and was not found in this study. Lastly, LBD was more prevalent in patients with triplications than in those with duplications. This suggests that LBD primarily occurs when the effects of *BHLHA9* overdosage are considerably elevated.

### Genomic basis of the Japanese founder copy number gains

The duplications/triplications were associated with the same fusion point and variable haplotype patterns. Since there was no sequence homology or low-copy repeats around the breakpoints, it is unlikely that such duplications/triplications were recurrently produced in different individuals by non-allelic homologous recombination (NAHR) [17,20]. Instead, it is assumed that a Japanese founder duplication took place in a single ancestor, and was spread with subsequent triplication and modification of the haplotype patterns.

The most likely genomic basis of the Japanese duplications/triplications is illustrated in Additional file 9. Notably, a 4 bp (GACA) microhomology was identified at the duplication fusion point (Figure 2B). A microhomology refers to two to five nucleotides common to the sequences of the two breakpoints, and is found as an overlapping sequence at the join point [16,19,20]. This suggests that the Japanese founder duplication was generated by replication-based mechanisms such as fork stalling and template switching (FoSTeS) and microhomology-mediated break-induced replication (MMBIR), because the presence of such a microhomology is characteristic of FoSTeS/MMBIR [17-20]. Indeed, such a simple tandem duplication with a microhomology can be produced by one time FoSTeS/MMBIR [17-20], although it could also be generated by non-homologous end-joining (NHEJ) [17]. Since the [A-14] haplotype was most prevalent on the duplicated/triplicated segments, it is inferred that a genomic rearrangement occurred in an ancestor with the [A-14] haplotype, yielding the founder duplication with the [A-14] + [A-14] haplotype. Furthermore, the presence of multiple stimulants for genomic rearrangements around the breakpoint on *YWHAE* intron 1 would have facilitated the generation of the founder duplication. In particular, non-B structures are known to stimulate the occurrence of both replication-based FoSTeS/MMBIR and double-strand breaks and resultant NHEJ [17,28,29], although the relative importance of each non-B DNA structure is largely unknown.

Subsequent triplication and haplotype modification can develop from the Japanese founder duplication through unequal interchromatid and interchromosomal recombinations [17,20]. Indeed, a tandem triplication with the [A-14] + [A-14] + [A-14] haplotype can be generated by unequal exchange between sister chromatids with the [A-14] + [A-14] haplotype, and various haplotype patterns are yielded by unequal interchromosomal exchanges involving the duplicated or triplicated segments. Furthermore, the haplotype variation would be facilitated by unequal exchanges between sister chromatids harboring duplications/triplications with various haplotype patterns and by the further unequal interchromosomal exchanges.

### Underlying factors for the phenotypic variability

**Figure 3:**
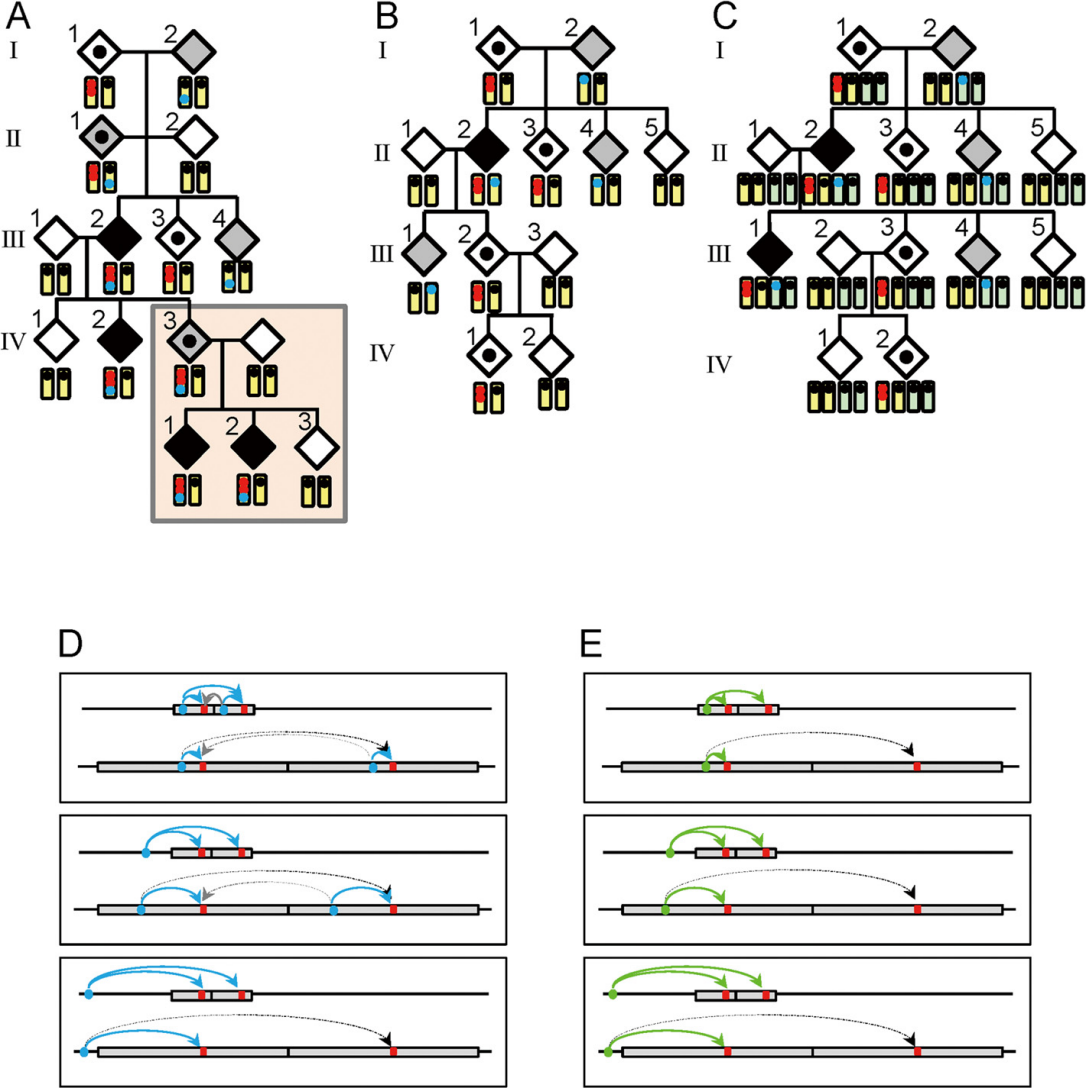
**Models for a modifier(s) and effects of the duplication size.** In models **A–C**, the yellow bars show chromosome 17, and the light green bars indicate other chromosomes. The two red dots represent the duplication at 17p13.3, and the blue dots indicate a putative modifier(s). Black painted diamonds represent limb malformation positive patients, dot-associated and gray painted diamonds indicate clinically normal carriers with the duplications and the modifier(s) respectively, and white painted diamonds denote clinically normal subjects without both the duplications and the modifier(s). **A**. This model assumes that co-existence of the duplication and a *cis*-acting modifier(s) causes limb malformation. If co-existence of the duplication and the *cis*-acting modifier(s) is associated with incomplete penetrance, this can explain all the transmission patterns observed to date, including the patient-to-carrier transmission and the presence of ≥ 2 affected children. **B**. This model postulates that the presence of a *cis*-acting modifier(s) on the normal chromosome 17 leads to limb malformation by enhancing the expression of the single *BHLHA9*, together with duplicated *BHLHA9* on the homologous chromosome. **C**. This model postulates that co-existence of the duplication at 17p13.3 and a modifier(s) on other chromosome causes limb malformation. In models **D–E**, the red bars represent *BHLHA9*, the blue circles indicate a physiological cis-regulatory element for *BHLHA9*, and the green circles indicate a non-physiological modifier(s) for *BHLHA9*. **D**. The physiological cis-regulatory element may be duplicated or non-duplicated, depending on its position relative to the size of the duplications. *BHLHA9* expression can be higher in small duplications than large duplications. **E**. The non-physiological modifier(s) can be transferred to various positions of the duplication positive chromosome 17, depending on the recombination places (see Model A). *BHLHA9* expression can be higher in small duplications than large duplications irrespective of the position of the modifier(s).

The duplications/triplications were accompanied by limb malformations with variable expressivity and incomplete penetrance. Although this may suggest the presence of a possible modifier(s) for the development of limb malformations, such a modifier(s) was not detected. In particular, while patient-to-carrier transmission of duplications/triplications was not identified in this study, even patient-to-carrier-to-patient transmission has been reported in three pedigrees [5,6,10]. Such transmission pattern with incomplete penetrance characterized by skipping of a generation is apparently inexplicable by assuming a modifier(s) interacting with *BHLHA9* or independent of *BHLHA9* on the duplication/triplication positive chromosome 17, on the normal chromosome 17, or on other chromosomes (Figure 3, Models A, B, and C, see also the legends in Figure 3).

In this regard, it is noteworthy that the development of limb malformations is obviously dependent on the size of genomic segment subjected to copy number gains. Actually, limb malformation has occurred in only one of 21 large duplications encompassing *BHLHA9* (average 1.55 Mb, mean 1.12 Mb) and in 29 of 80 small duplications encompassing *BHLHA9* (average 244 kb, mean 263 kb) (*P* = 5.9 × 10^−3^) [8]. Consistent with this, the patients with large and small duplications were ascertained primarily due to developmental retardation and limb malformation, respectively [8]. It is likely that a physiological cis-regulatory element for *BHLHA9* (e.g., an enhancer) can frequently but not invariably work on both of the duplicated *BHLHA9* when the duplication size is small but is usually incapable of working on duplicated *BHLHA9* when the duplication size is large, probably because of the difference in the chromatin structure (see Model D in Figure 3). Similar findings have also been reported in other genes. For example, small (~150 kb) and relatively small (600–800 kb) duplications involving a putative testis-specific enhancer(s) for *SOX9* have caused 46,XX testicular and ovotesticular disorders of sex development respectively, whereas large duplications (~2 Mb) involving the enhancer(s) have permitted normal ovarian development in 46,XX individuals [30].

Thus, a plausible explanation may be that a range of limb malformations emerge when the effects of *BHLHA9* overdosage exceed the threshold for the development of SHFM, SHFLD, or GWC, depending on the conditions of other genetic and environmental factors including the size of duplications/triplications as an important but not definitive factor. One may argue that this notion is inconsistent with the apparent anticipation phenomenon that is suggested by the rare patient-to-carrier transmission and the frequent carrier-to-patient transmission of the duplications/triplications, because no specific factor(s) exaggerating the development of limb malformations is postulated in the next generation. However, the skewed transmission pattern would primarily be ascribed to ascertainment bias rather than anticipation [31]. Indeed, while clinically normal parents of disease positive children would frequently be examined for the underlying genetic factor(s) of the children, clinically normal children born to disease positive parents would not usually be studied for such factor(s), as exemplified in this study. Similarly, the frequent patient-to-patient transmission of the duplications/triplications would also be ascribed to ascertainment bias, because molecular studies would preferentially be performed in such families. Nevertheless, the apparently autosomal dominant inheritance pattern of limb malformations in several families may still suggest the relevance of a non-physiological *cis*-acting modifier(s) (see Models A and E in Figure 3). It is possible that such a modifier(s), once transferred onto the duplication/triplication positive chromosome 17, is usually co-transmitted with the duplications/triplications, leading to a specific condition in which the effects of *BHLHA9* overdosage frequently but not invariably exceed the threshold for the development of limb malformations in offsprings with the duplications/triplications.

### Remarks

Several matters should be pointed out in the present study. First, in contrast to diverse duplication sizes in non-Japanese populations [5-9], the size of the genomic segment subjected to duplications/triplications was identical in this study. Since families 1–27 were derived from various places of Japan, there is no selection bias in terms of a geographic distribution. Rather, since the small duplications/triplications identified in this study were not associated with developmental retardation, it is likely that they spread throughout Japan primarily via carriers with normal fitness and were found via patients with limb malformations. Obviously, this notion does not exclude the possible presence of other types of duplications/triplications at 17p13.3 in Japan. Second, except for the duplications/triplications at 17p13.3, we could reveal a homozygous *WNT10B* mutation (SHFM6) only in a single SHFM family and chromosome 10q24 duplications (SHFM3) only in three SHFM families. Thus, underlying factors are still unknown in the remaining 20 families, although tiny deletions and/or duplications affecting the known SHFM loci might have been overlooked because of the low resolution of the array. In addition, although all the probands had a normal karyotype, there might be cryptic translocations and/or inversions involving the known SHFM loci. Third, no deletion of *BHLHA9* was identified in the 51 probands and in the 200 control subjects. This argues against the relevance of *BHLHA9* haploinsufficiency to limb malformations, and coincides with the Japanese founder duplication being produced by a replication-mediated mechanism rather than an interchromatid/interchromosomal (but not an intrachromatid) NAHR that can lead to both deletions and duplications as a mirror image [17]. Furthermore, it remains to be determined (i) whether gain-of-function mutations (and possibly loss-of-function mutations as well) of *BHLHA9* are identified in patients with limb malformations, (ii) whether duplications/triplications involving *BHLHA9* underlie limb malformations other than SHFM, SHFLD, and GWC, and (iii) whether *BHLHA9*-containing duplications/triplications are also the most frequent underlying factors for limb malformations in non-Japanese populations.

## Conclusions

The results imply that (i) duplications/triplications involving *BHLHA9* at chromosome 17p13.3 constitute a strong susceptibility factor for the development of a range of limb malformations including SHFM, SHFLD, and GWC; (ii) the Japanese founder duplication was generated by a replication-based mechanism and spread with subsequent triplication and haplotype modification through recombination-based mechanisms; and (iii) clinical variability appears to be due to multiple factors including the size of duplications/triplications. Thus, the present study provides useful information on the development of limb malformations.

## Additional files

Additional file 1: Table S1. Primers utilized in this study. Additional file 2: Figure S1. Real-time PCR analysis. Additional file 3: Figure S2.
*D17S1174* analysis in 200 Japanese control subjects, showing discontinuous distribution of the CA repeat numbers, as observed in the Japanese families with limb malformations. Additional file 4: Table S2.
*In silico* analysis for specific structures around the breakpoint-flanking regions and control regions. Additional file 5: Table S3. Phenotypes in patients/subjects with increased copy number of *BHLHA9*. Additional file 6: Figure S3. Genomic region encompassing *BHLHA9* examined in this study. Additional file 7: Table S4. Polymorphism analysis of rs3951819 (A/G SNP) in *BHLHA9*. Additional file 8: Table S5. Polymorphism analysis of rs34201045 (4 bp insertion) in *TP63*. Additional file 9: Figure S4. Genomic basis of the Japanese founder copy number gain.

## Abbreviations

AER: Apical ectodermal ridge
CEP17: Centromere of chromosome 17
CGH: Comparative genomic hybridization
Dup: Duplication
FoSTeS: Fork stalling and template switching
GWC: Gollop-Wolfgang complex
L: Lower
LBD: Long bone defect
MMBIR: Microhomology-mediated break-induced replication
NAHR: Non-allelic homologous recombination
N.E.: Not examined
NHEJ: Non-homologous end-joining
qPCR: Quantitative real-time PCR
SF: Split foo17)t
SH: Split hand
SHFLD: SHFM with long bone deficiency
SHFM: Split-hand/foot malformation
Trip: Triplication
U: Upper
