# Supplementary material for: Japanese founder duplications/triplications involving BHLHA9 are associated with split-hand/foot malformation with or without long bone deficiency and Gollop-Wolfgang complex
Source: Orphanet J Rare Dis. 2014 Oct 21;9:125. doi: 10.1186/s13023-014-0125-5 (PMC4205278; doi:10.1186/s13023-014-0125-5)
Supplement: Additional file 1: Table S1. — Primers utilized in this study. [file 13023_2014_125_MOESM1_ESM.pdf]

Table S1 Primers utilized in this study.

		Forward (5'→3')	Reverse (5'→3')	AT	PS	Reference
DLX5 sequencing	Exon 1-1	CCCAGACATCACAGAGCCTT	GCTGAAGACTCGGGCAAAGT	60	651	This report
	Exon 1-2	GTTTGACAGAAGGGTCCCCA	CTCCACAGGGCTCTCCTACT	60	545	This report
	Exon 2	CTATTTGAGGCCCTTCCGCA	CTTGCTTTCAACCCGCGAAA	65	542	This report
	Exon 3	CCCAGAGGTCCGAAGATGC	TGCCTTGTTGGATCTCTGCA	63	875	This report
TP63 sequencing	Exon 1	TCCCGGCTTTATATCTATATATAC	GACACATTCATAATACACAAGGCAC	60	220	1
	Exon 2	TCCACTTGGGTTTTCATGATAGAG	GTAAGCAATATTTTGACCACCCAC	60	300	1
	Exon 3	GCTTGTTGTTAACAACAGCATG	GAAAAGACAGGTTTAACAGAGC	60	281	1
	Exon 3-1	GAGAGAGAGGGACTTGAGTTCTG	GACCGAGAACCGCAAATACG	60	261	1
	Exon 4	GGCTAATATTGGGGTTTCTGG	GGGGTCAGGTATGTGCGT	60	321	1
	Exon 5	CCTGTTGGTTCTCTCCTTCCT	TCAAACAAAAATGCCCCACAG	60	306	1
	Exon 6	CCACCAACATCCTGTTCATGC	GTCTACTCAGTCCATAGAGGTGTTG	60	257	1
	Exon 7	GAAGGAACAACGTCAGTTTAAACCC	AAAGCAGCCACGATTTCACTTTGCC	60	245	1
	Exon 8	GTGGTAGATCTTCAGGGGACTTTC	CCAACATCTGGAGAAGATTC	60	263	1
	Exon 9	GCTTTAGAAGTGTTCCCAGG	ACACCTCCTTTCCCATTGTC	60	237	1
	Exon 10	TGAGGATTGACCACACTTCTAAC	CATCAATCACCCTATTGCTGATC	60	287	1
	Exon 11	TGCTCACCATTATTTCCATGTTTGTC	TCACAGAGTCTTGTCCTAAGC	60	257	1
	Exon 12	GGACTATAACAGTATCCGCCC	CAAGATGGACCACTGGGATG	60	294	1
	Exon 13	CTTATCTCGCCAATGCAGTTGG	AACTACAAGGCGGTTGTCATCAG	60	241	1
	Exon 14	GGGAATGATAGGATGCTGTGG	AAGATTAAGCAGGAGTGCTT	60	449	1
	Exon 15	GATGAAGTCCTAGGCCTTC	GGAAATACAACACACACACT	60	205	1
WNT10B sequencing	Exon 2	GTGTCTGATTGGGCAAGGTT	CTCATTGCTTAGAGCCCTGG	60	491	1
	Exon 3	GGAGAGTTGGAGGGGTCTG	GAAACCATCCCTTCCCGC	64	405	1
	Exon 4	TGCCTGTCAACCTTACCTCC	TAACCAGGCCTCAAAAGCTG	60	470	1
	Exon 5	TGTGCCTCTGTGTTCTGTCC	GAAATCAGAGCAAAGGGCTG	60	598	1
BHLHA9 sequencing	Exon 1-1	AGAGGGCAGAGGGCAGAG	CGTGGCACTCCAGGTGTC	65	469	This report
	Exon 1-2	CAAGAGGCTCTCCAAGATCG	CCAAGACGCCTTTGCAGT	60	498	This report
	Exon 1-3	GGCTGGCAGCTGGACTATAA	AGGATCCTGAGCCTTTCTGG	60	235	This report
7,406 bp sequencing		CGTGATTTCTCCACTGTTACCTGTTTGTTTCTTT	CCACATATAAGTCATCTTGGAGAATGGACACGAT	66	7,406	This report
FISH probe		CAGAAGTAAATACATACACTCTGCTTCCTAATGC	ATCCAAGTTGAGATGTAAAATACACACAAGACAT	66	8259	This report
Fusion point analysis	P1/P2	TCATTGTATGGTAATAGCCTAGACACAGATCC	CCGATATCGTGTTGTTTTATAGTGTTTACCTG	66	2195	This report
	P3/P4	AATGTGGTTGAGACCCAGGC	CACACTGGATGGGAAGGACC	60	418	This report
	P5/P6	CTCAGCCAGGTGAAGAGGAC	CGAGGATCCTTTTGTTTGCT	60	214	This report
	RNaseP	CCTGGTACATGCCACTGATG	AGTGTAGAGGGCAAGCCAGA	60	213	2
Microsatellite analysis	*D17S1174*	AGGGGGAATCGCTTAAACCC	TTGGAGACATGGCAGGGAAC	60	387	This report
Southern blot analysis	Probe 1	GACGGATAACAAGGACCGTG	TTTGGAGAGTTGATACAGCG	58	611	This report
	Probe 2	CTTATTCCAGAGCCTACACAAC	CCTTTTTCTGGAGAATATACTC	55	241	This report
	Probe 3	CACTCTGCTTCCTAATGCC	TTTTGGTTGGGCCTGACTC	58	504	This report
	Probe 4	AGGCTGTGACACAGACCAC	GCCCCCTTACTTCTTTCAG	58	447	This report
	Probe 5	GTCAGGAGGCTACTCTCTCAC	AGAGTAGTCCCTGGCACGAG	58	533	
Methylation analysis		CCTGGGAGAGAAGGAGGCTCAGG	TGGGCATTTTCTTTGCTTTTACTGACC		514	This report
Mouse expression study		CAAGAGGCTCTCCAAGATCG	CAGCATATGCCCTCAGATCA			This report

AT: annealing temperature (°C); and PS: product size (bp).

References

1. Ugur SA et al.: Hum Mol Genet 2008, **17:**2644–2653.

2. Wu X et al.: Breast Cancer Res 2012, **14:** R73.
