# Supplementary material for: Japanese founder duplications/triplications involving BHLHA9 are associated with split-hand/foot malformation with or without long bone deficiency and Gollop-Wolfgang complex
Source: Orphanet J Rare Dis. 2014 Oct 21;9:125. doi: 10.1186/s13023-014-0125-5 (PMC4205278; doi:10.1186/s13023-014-0125-5)
Supplement: Additional file 2: Figure S1. — Real-time PCR analysis. [file 13023_2014_125_MOESM2_ESM.pdf]

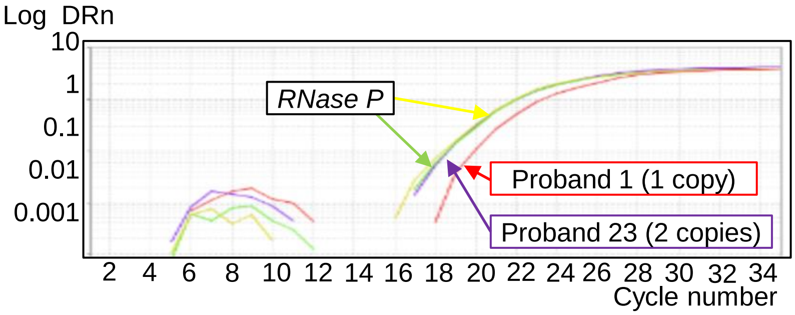


**Figure S1.** Real-time PCR analysis. Shown are the raw data indicating the presence of a single copy and two copies of the fusion point in the proband 1 and the proband 23, respectively. *RNaseP* has been utilized as an internal control.
