# Supplementary material for: Japanese founder duplications/triplications involving BHLHA9 are associated with split-hand/foot malformation with or without long bone deficiency and Gollop-Wolfgang complex
Source: Orphanet J Rare Dis. 2014 Oct 21;9:125. doi: 10.1186/s13023-014-0125-5 (PMC4205278; doi:10.1186/s13023-014-0125-5)
Supplement: Additional file 3: Figure S2. — D17S1174 analysis in 200 Japanese control subjects, showing discontinuous distribution of the CA repeat numbers, as observed in the Japanese families with limb malformations. [file 13023_2014_125_MOESM3_ESM.pdf]

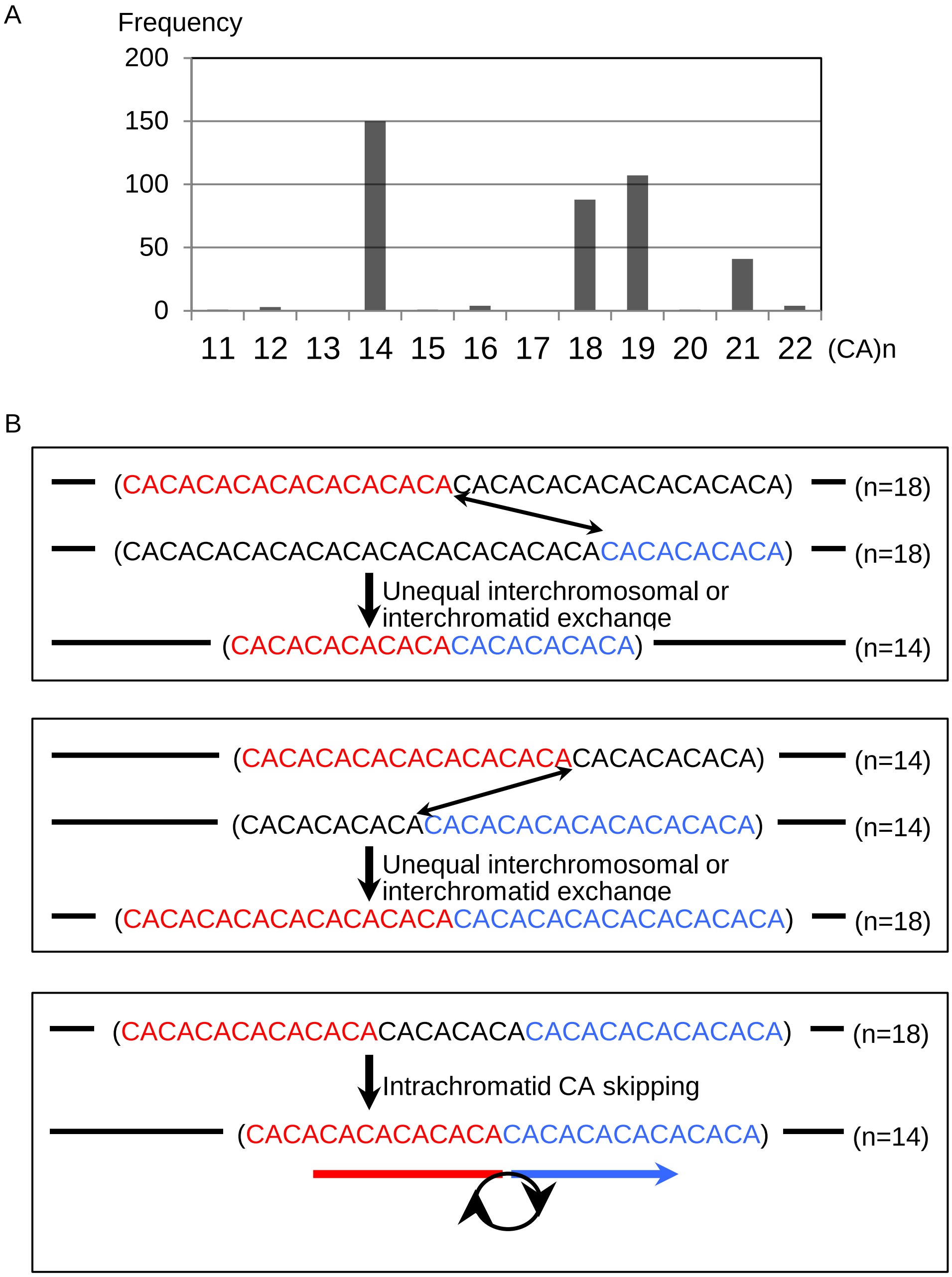


**Figure S2.** *D17S1174* analysis in 200 Japanese control subjects, showing discontinuous distribution of the CA repeat numbers, as observed in the Japanese families with limb malformations.

A. Distribution of CA repeat numbers. Although a slippage phenomenon would account for the CA repeat numbers of 19 and 18, it does not explain the discontinuous distribution.

B. Schematic representation of the mechanisms leading to the discontinuous distribution. Unequal interchromosomal/interchromatid exchanges affecting the CA repeat stretch can lead to non-continuous extension and shortening of the CA repeat number, and intrachromatid rearrangements affecting the CA repeat stretch can result in non-continuous shortening of the CA repeat number.
