# Supplementary material for: Japanese founder duplications/triplications involving BHLHA9 are associated with split-hand/foot malformation with or without long bone deficiency and Gollop-Wolfgang complex
Source: Orphanet J Rare Dis. 2014 Oct 21;9:125. doi: 10.1186/s13023-014-0125-5 (PMC4205278; doi:10.1186/s13023-014-0125-5)
Supplement: Additional file 4: Table S2. — In silico analysis for specific structures around the breakpoint-flanking regions and control regions. [file 13023_2014_125_MOESM4_ESM.pdf]

Table S2 *In silico* analysis for specific structures around the breakpoint-flanking regions and control regions

	Centomeric breakpoint (*YWHAE* intron 1)	Telomeric breakpoint (*ABR* intron 1)	Control regions (ch. 17) (n =48)
GC content (%)	41.0	60.5	44.8
Palindrome	1	0	0.21
Non-B (non-canonical) DNA structure			
Direct repeat	3	0	0.23
Inverted repeat, cruciform motif	3	0	1.67
Mirror repeat	5	0	0.33
A-phased repeat	0	0	0.14
G-quadruplex repeat	0	0	0.10
Short tandem repeat	4	0	0.71
Z-DNA motif	0	0	0.10
Rearrangement-associated sequence motif			
Homologous recombination hotspot motif (CCNCCNTNNCCNC)	0	0	0.06
DNA polymerase A frameshift hotspot 1 (TCCCCC)	0	0	0.46
DNA polymerase B frameshift hotspot 1 (ACCCWR)	1	1	1.40
Translin binding site 2 (GCCCWSSW)	1	3	0.65
Consensus scaffold attachment region 4 (TWWTDTTWWW)	0	0	3.67
Murine parvovirus recombination hotspot (CTWTTY)	7	2	2.31
Murine MHC recombination hotspot (GAGRCAGR)	0	0	0.81
Duplication-associated motif (CTSAGYTTTT)	0	1	0.02
Duplication-associated motif (WSCAGGNAYWWTTCC)	0	0	0
Duplication-associated motif (ATTTCTYCAGYNYTGGATHT)	0	0	0
Rearrangement-associated tri/tetranucleotide			
Minimal primase recognition site of prokaryote (CTG/CAG)	28	39	29.25
Recombination-associated tetranucleotide (CCTG)	7	14	8.75

Except for CG content, the number of each motif is shown for the centromeric and the telomeric breakpoints of 600 bp long.
For control regions, the mean number of each motif is shown.
