# Supplementary material for: Japanese founder duplications/triplications involving BHLHA9 are associated with split-hand/foot malformation with or without long bone deficiency and Gollop-Wolfgang complex
Source: Orphanet J Rare Dis. 2014 Oct 21;9:125. doi: 10.1186/s13023-014-0125-5 (PMC4205278; doi:10.1186/s13023-014-0125-5)
Supplement: Additional file 5: Table S3. — Phenotypes in patients/subjects with increased copy number of BHLHA9. [file 13023_2014_125_MOESM5_ESM.pdf]

Table S3 Phenotypes in patients/subjects with increased copy number of *BHLHA9*

Patient			Ectrodactyly		Long bone deficiency		Phenotype
Family	Subject	Sex	Hands	Feet	Tibia	Femur	
1	II-1	M	+/+	+/+	+/+	+/–	GWC
2	II-1	F	+/+	+/–	+/–	–/–	SHFLD
	II-2	M	+/+	+/+	+/+	–/–	SHFLD
3	I-1	M	–/+	–/–	+/–	–/–	SHFLD
	II-2	F	+/+	–/+	–/–	–/–	SHFM
	II-3	F	+/–	–/–	–/–	–/–	SHFM
4	II-1	M	+/–	–/–	+/–	–/–	SHFLD
5	II-1	F	–/+	–/–	–/+	–/–	SHFLD
6	II-1	M	+/–	+/+	+/+	–/–	SHFLD
7	II-1	M	+/–	–/+	–/–	–/–	SHFM
	II-3	F	+/–	–/–	–/–	–/–	SHFM
	III-2	M	+/+	–/–	–/–	–/–	SHFM
	IV-1	F	+/+	–/–	–/–	–/–	SHFM
8	I-1	M	+/–	+/–	–/–	–/–	SHFM
	II-1	M	+/–	+/–	–/–	–/–	SHFM
9	I-1	M	+/+	–/–	–/–	–/–	SHFM
	II-2	M	+/+	–/–	–/–	–/–	SHFM
10	I-2	F	+/+	–/–	–/–	–/–	SHFM
	II-1	F	+/–	–/–	–/–	–/–	SHFM
	II-2	F	–/+	–/–	–/–	–/–	SHFM
11	II-1	F	+/–	–/–	–/–	–/–	SHFM
12	II-1	M	+/–	–/–	–/–	–/–	SHFM
	II-3	M	–/+	–/–	–/–	–/–	SHFM
13	II-3	F	+/+	–/–	–/–	–/–	SHFM
14	II-1	M	–/+	–/–	–/–	–/–	SHFM
15	II-2	M	+/–	–/–	–/–	–/–	SHFM
16	II-3	M	+/–	–/–	–/–	–/–	SHFM
17	II-2	F	+/–	–/–	–/–	–/–	SHFM
18	II-1	M	+/+	+/+	–/–	–/–	SHFM
19	II-2	F	+/+	–/–	–/–	–/–	SHFM
20	II-1	M	+/+	+/–	–/–	–/–	SHFM
21	II-1	F	+/+	–/–	–/–	–/–	SHFM
22	II-1	M	+/+	–/–	–/–	–/–	SHFM
23	II-1	M	–/–	+/–	+/+	+/–	GWC
	II-2	M	+/+	+/–	+/–	–/–	SHFLD
24	II-1	M	+/–	+/–	+/–	–/–	SHFLD
	III-1	M	+/+	+/–	+/+	–/–	SHFLD
25	III-1	M	+/+	+/+	+/+	–/–	SHFLD
	III-2	F	+/+	–/–	–/–	–/–	SHFM
26	II-2	M	–/+	–/–	+/+	–/–	SHFLD
27	I-2	F	+/+	+/+	–/–	–/–	SHFM
	II-3	M	–/+	–/+	–/–	–/–	SHFM

Right/Left.

Patient numbers correspond to those of Fig. 1.

Patient 3-II-2 has ventricular septal defect and aortic coarctation.

Patient 13-II-1 has hypospadias.

Patient 15-II-2 has polydactyly of the left foot.
