# Supplementary material for: Japanese founder duplications/triplications involving BHLHA9 are associated with split-hand/foot malformation with or without long bone deficiency and Gollop-Wolfgang complex
Source: Orphanet J Rare Dis. 2014 Oct 21;9:125. doi: 10.1186/s13023-014-0125-5 (PMC4205278; doi:10.1186/s13023-014-0125-5)
Supplement: Additional file 6: Figure S3. — Genomic region encompassing BHLHA9 examined in this study. [file 13023_2014_125_MOESM6_ESM.pdf]

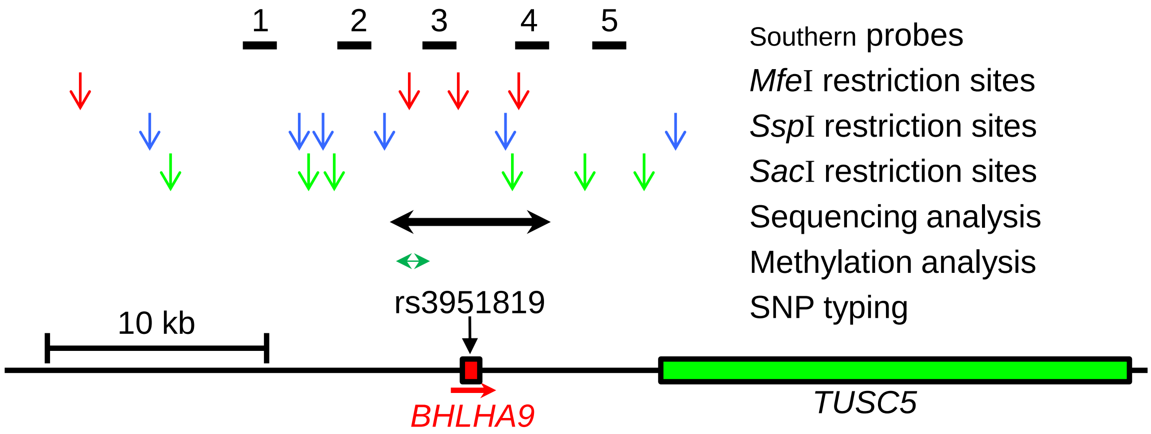

Figure S3. Genomic region encompassing *BHLHA9* examined in this study. According to GRCh37/hg19, the physical positions from the 17p telomere are (1) *BHLHA9* coding region: 1,173,853–1,174,754 bp; (2) rs3951819: 1,174,094 bp; (3) CpG rich region examined by the methylation analysis: 1,171,460 – 1,1719,73 bp; (4) a 7,406 region subjected to sequence analysis: 1,170,107–1,177,512 bp; (5) *Sac*I restriction sites: 1,158,741–1,158,746 bp, 1,165,510–1,165,515 bp, 1,167,455–1,167,460 bp, 1,170,783–1,170,788 bp, 1,174,693–1,174,698 bp, and 1,183,075–1,183,080 bp; (6) *Ssp*I restriction sites: 1,160,981–1,160,986 bp, 1,166,006–1,166,011 bp, 1,167,828–1,167,833 bp, 1,175,758–1,175,763 bp, 1,177,578–1,177,583 bp, and 1,180,542–1,180,547 bp; (7) *Mfe*I restriction sites: 1,148,976–1,148,981 bp, 1,171,403–1,171,408 bp, 1,172,928–1,172,933 bp, and 1,176,035–1,176,040 bp, and (8) five Southern probe segments: 1,163,343–1,163,953 bp, 1,168,469–1,168,709 bp, 1,172,415–1,172,918 bp, 1,176,048–1,176,494 bp, and 1,179,204–1,179,736 bp.
