# Supplementary material for: Japanese founder duplications/triplications involving BHLHA9 are associated with split-hand/foot malformation with or without long bone deficiency and Gollop-Wolfgang complex
Source: Orphanet J Rare Dis. 2014 Oct 21;9:125. doi: 10.1186/s13023-014-0125-5 (PMC4205278; doi:10.1186/s13023-014-0125-5)
Supplement: Additional file 7: Table S4. — Polymorphism analysis of rs3951819 (A/G SNP) in BHLHA9. [file 13023_2014_125_MOESM7_ESM.pdf]

Table S4 Polymorphism analysis of rs3951819 (A/G SNP) in *BHLHA9*

	Polymorphism analysis					Statistical analysis (*P*-value)			
	Patients				Carriers	SHFM vs. SHFLD	Carriers vs. Patients		
	Total (n=27)	SHFM (n=15)	SHFLD (n=10)	GWC (n=2)	(n=17)		Total	SHFM	SHFLD
<Allele frequency*>									
A	16	7	7	2	11	0.23	0.49	0.25	0.56
G	11	8	3	0	6				
<Genotype frequency>									
AA/A	6	4	1	1	4	…	…	…	…
AA/G	2	2	0	0	3	…	…	…	…
AG/A	5	2	3	0	1	…	…	…	…
AG/G	5	4	1	0	3	…	…	…	…
AAA/A	4	0	3	1	4	…	…	…	…
AAA/G	1	0	1	0	0	…	…	…	…
AAG/A	0	0	0	0	2	…	…	…	…
AAG/G	2	1	1	0	0	…	…	…	…
AGA/A	0	0	0	0	0	…	…	…	…
AGA/G	0	0	0	0	0	…	…	…	…
AGG/A	1	1	0	0	0	…	…	…	…
AGG/G	1	1	0	0	0	…	…	…	…

* On the duplications/triplications negative normal chromosome 17.

The 95% confidence intervals for odds ratios include 1.0 in all the comparisons (not shown).

P-values have not been caluculated for GWC, because of the small patient number.

The allele frequency in the Japanese population is 60% for A and 40% for G
