# Supplementary material for: Japanese founder duplications/triplications involving BHLHA9 are associated with split-hand/foot malformation with or without long bone deficiency and Gollop-Wolfgang complex
Source: Orphanet J Rare Dis. 2014 Oct 21;9:125. doi: 10.1186/s13023-014-0125-5 (PMC4205278; doi:10.1186/s13023-014-0125-5)
Supplement: Additional file 8: Table S5. — Polymorphism analysis of rs34201045 (4 bp insertion) in TP63. [file 13023_2014_125_MOESM8_ESM.pdf]

Table S5 Polymorphism analysis of rs34201045 (4 bp insertion) in *TP63*

	Polymorphism analysis					Statistical analysis (*P*-value)			
	Patients				Carriers	SHFM vs. SHFLD	Carriers vs. Patients		
	Total (n=27)	SHFM (n=17)	SHFLD (n=8)	GWC (n=2)	(n=15)		Total	SHFM	SHFLD
<Allele frequency>									
Insertion (–)	23	15	7	1	13	0.61	0.62	0.58	0.61
Insertion (+)	31	19	9	3	17				
<Genotype frequency>									
Insertion (–)/(–)	5	3	2	0	2	0.89	0.72	0.73	0.96
Insertion (–)/(+)	13	9	3	1	8				
Insertion (+)/(+)	9	5	3	1	3				
Insertion (–)/(–)	5	3	2	0	2	0.62	0.59	0.67	0.55
Insertion (–)/(+) plus Insertion (+)/(+)	22	14	6	2	11				
Insertion (–)/(–) plus Insertion (–)/(+)	18	12	5	1	10	0.68	0.39	0.43	0.48
Insertion (+)/(+)	9	5	3	1	3				

The genotyoing data satisfy the Hardy-Weinberg equilibrium (Fisher's exact test with one degree of freedom).
The (+) and the (–) symbols represent the presence and absence of the 4 bp insertion, respectively.
The 95% confidence intervals for odds ratios include 1.0 in all the comparisons (not shown).
P-values have not been caluculated for GWC, because of the small patient number.
The allele frequency in 100 Japanese control subjects is 44% for Insertion (–) and 56% for Insertion (+) (our data).
