# Supplementary material for: Japanese founder duplications/triplications involving BHLHA9 are associated with split-hand/foot malformation with or without long bone deficiency and Gollop-Wolfgang complex
Source: Orphanet J Rare Dis. 2014 Oct 21;9:125. doi: 10.1186/s13023-014-0125-5 (PMC4205278; doi:10.1186/s13023-014-0125-5)
Supplement: Additional file 9: Figure S4. — Genomic basis of the Japanese founder copy number gain. [file 13023_2014_125_MOESM9_ESM.pdf]

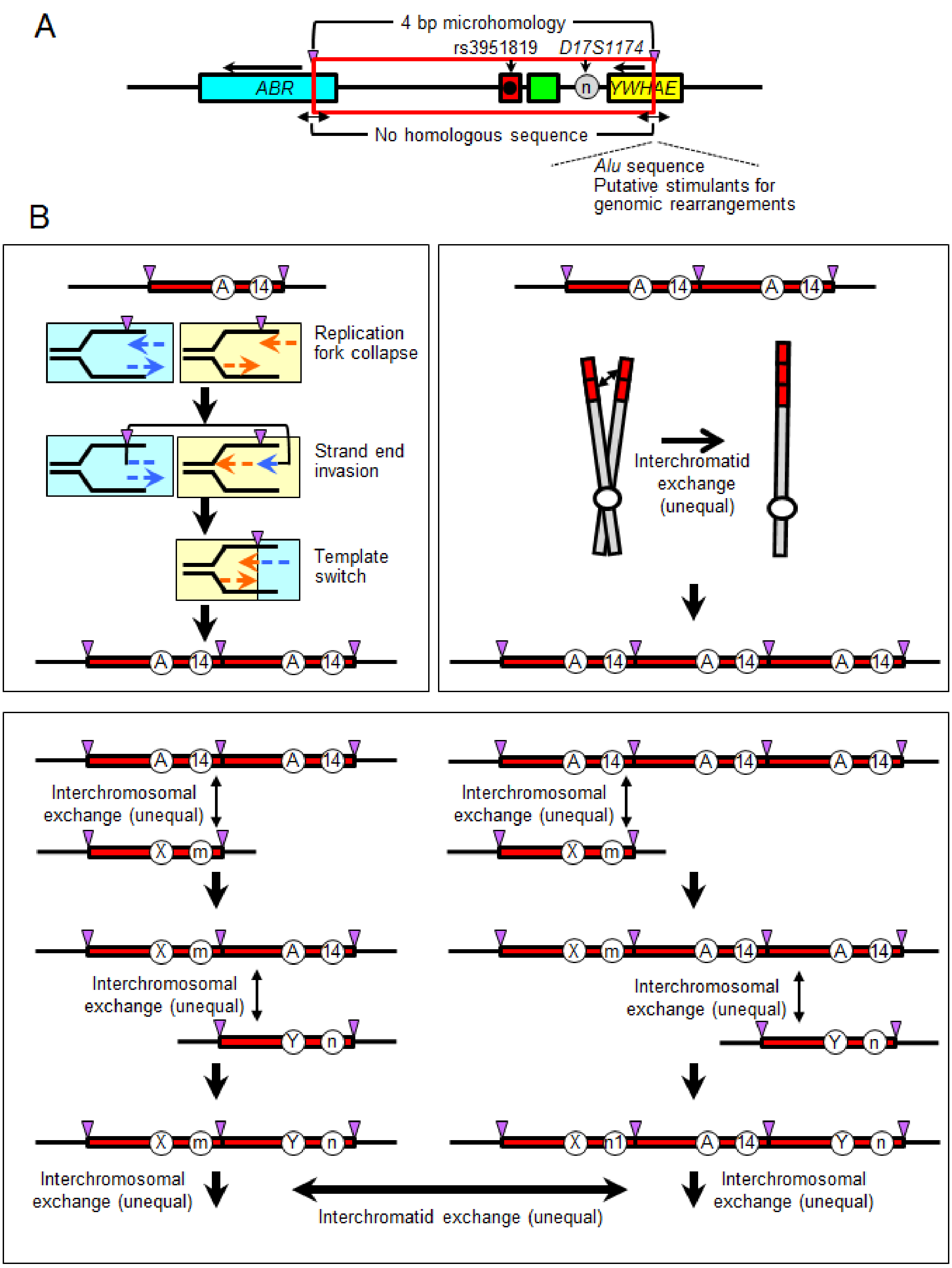

Figure S4. Genomic basis of the Japanese founder copy number gain.

A. Structure of the ~ 200 kb segment region subjected to duplications/triplications. The red open rectangle indicates the duplicated/triplicated segment. The red and green boxes denote *BHLHA9* and *TUSC5*, respectively. *ABR* and *YWHAE* are transcribed in the same direction (arrows). The inverted purple triangles show the 4 bp microhomology. The rs3951819 (A/G SNP) and *D17S1174* (CA repeat number) reside within the ~ 200 kb segment.

B. Upper left part: Formation of the Japanese founder duplication with the [A-14]+[A-14] haplotype pattern in an ancestor with the [A-14] haplotype through FoSTeS/MMBIR (1 ×). The blue and the yellow boxes represent the *ABR* and *YWHAE* sequences harboring the fusion point. The leading nascent strand fork of *ABR* invades the strand fork of *YWHAE* via the 4 bp microhomology and primes its own synthesis, thereby producing a simple duplication.

Upper right part: Generation of triplication through unequal exchange between duplication positive sister chromatids.

Lower part: Development of various haplotype patterns through unequal interchromosomal exchanges. X & Y: A or G; and m & n: the CA repeat number.
